# Intensive care antibiotic consumption and resistance patterns: a cross-correlation analysis

**DOI:** 10.1186/s12941-017-0251-8

**Published:** 2017-11-13

**Authors:** Luminita Baditoiu, Carmen Axente, Diana Lungeanu, Delia Muntean, Florin Horhat, Roxana Moldovan, Elena Hogea, Ovidiu Bedreag, Dorel Sandesc, Monica Licker

**Affiliations:** 1 Epidemiology Department, “Victor Babes” University of Medicine and Pharmacy, Timisoara, Romania; 2 Microbiology Department, “Victor Babes” University of Medicine and Pharmacy, 16 Victor Babes, Timisoara, Romania; 3 Department of Functional Sciences, Centre for Modelling Biological Processes and Data Analysis, “Victor Babes” University of Medicine and Pharmacy, Timisoara, Romania; 4 “Pius Branzeu” Emergency Clinical County Hospital, Timisoara, Romania; 5 Regional Center of Public Health Timisoara, Timisoara, Romania; 6 “Victor Babes” Clinical Hospital, Timisoara, Romania; 7 Anesthesiology and Intensive Care Department, “Victor Babes” University of Medicine and Pharmacy, Timisoara, Romania

**Keywords:** ICU, Antibiotic consumption, Defined daily dose, Multidrug resistance, Regression modeling

## Abstract

**Background:**

Over recent decades, a dramatic increase in infections caused by multidrug-resistant pathogens has been observed worldwide. The aim of the present study was to investigate the relationship between local resistance bacterial patterns and antibiotic consumption in an intensive care unit in a Romanian university hospital.

**Methods:**

A prospective study was conducted between 1st January 2012 and 31st December 2013. Data covering the consumption of antibacterial drugs and the incidence density for the main resistance phenotypes was collected on a monthly basis, and this data was aggregated quarterly. The relationship between the antibiotic consumption and resistance was investigated using cross-correlation, and four regression models were constructed, using the SPSS version 20.0 (IBM, Chicago, IL) and the R version 3.2.3 packages.

**Results:**

During the period studied, the incidence of combined-resistant and carbapenem-resistant *P. aeruginosa* strains increased significantly [(gradient = 0.78, R^2^ = 0.707, p = 0.009) (gradient = 0.74, R^2^ = 0.666, p = 0.013) respectively], mirroring the increase in consumption of β-lactam antibiotics with β-lactamase inhibitors (piperacillin/tazobactam) and carbapenems (meropenem) [(gradient = 10.91, R^2^ = 0.698, p = 0.010) and (gradient = 14.63, R^2^ = 0.753, p = 0.005) respectively]. The highest cross-correlation coefficients for zero time lags were found between combined-resistant vs. penicillins consumption and carbapenem-resistant *P. aeruginosa* strains vs. carbapenems consumption (0.876 and 0.928, respectively). The best model describing the relation between combined-resistant *P. aeruginosa* strains and penicillins consumption during a given quarter incorporates both the consumption and the incidence of combined-resistant strains in the hospital department during the previous quarter (multiple R^2^ = 0.953, *p* = 0.017). The best model for explaining the carbapenem resistance of *P. aeruginosa* strains based on meropenem consumption during a given quarter proved to be the adjusted model which takes into consideration both previous consumption and incidence density of strains during the previous quarter (Multiple R^2^ = 0.921, p = 0.037).

**Conclusions:**

The cross-correlation coefficients and the fitted regression models provide additional evidence that resistance during the a given quarter depends not only on the consumption of antibacterial chemotherapeutic drugs in both that quarter and the previous one, but also on the incidence of resistant strains circulating during the previous quarter.

**Electronic supplementary material:**

The online version of this article (10.1186/s12941-017-0251-8) contains supplementary material, which is available to authorized users.

## Background

Over recent decades, a dramatic increase in infections caused by multidrug-resistant (MDR) pathogens has been observed worldwide, with urgent need for new approaches to antibiotic therapy, in parallel with discovery of new antimicrobial agents [1–6]. Intensive care units (ICU) where over 60% of patients receive antibiotic treatment, especially broad-spectrum antimicrobials, and where highly invasive therapeutic interventions are involved, represent the epicentres for the emergence and spread of MDR strains in tertiary medical units [7]. This emergence is not only influenced by the consumption of antimicrobials but also by the clonal spread of strains, multiple human or environmental infection sources, together with MDR infection control strategies and screening procedures upon hospital admission.

Studies concerning the correlation between antibiotic consumption and the increasing resistance of bacterial strains provide different and sometimes divergent results, possibly due to the peculiarities of certain resistance phenotypes and antibiotic prescribing practices. But all the studies affirm the need to monitor antibiotic consumption, both locally and nationally [4, 8].

In Europe and other areas, the prevalence of extended spectrum β-lactamase (ESBL) producing organisms has increased, especially for *Klebsiella pneumoniae* strains [9–11]. The incidence of infections caused by these strains strongly correlates with the use of ceftazidime, imipenem, and amoxicillin/clavulanic acid [12]. In other studies, the prevalence of ESBL *K. pneumoniae* strains is associated with the use of ciprofloxacin or third-generation cephalosporins [13].

Regarding non-fermentative germs, positive correlations have been identified between previous long-term administration of β-lactam antibiotics or carbapenems and pan-drug-resistant *Pseudomonas aeruginosa* infections [12, 14]. Positive correlations related to *P. aeruginosa* were also identified between consumption and the occurrence of imipenem resistance during the same and the following quarter, between meropenem usage and MDR *P. aeruginosa* strains, and between consumption and resistance to ciprofloxacin [13, 15, 16].

The increased use of carbapenems, which are among the most effective classes of antimicrobials against MDR Gram-negative bacilli (GNB), has been associated with the emergence of carbapenem-resistant *Enterobacteriaceae* or *A. baumannii* [1, 2, 16–18].

*Pseudomonas aeruginosa*, an ubiquitary germ in nosocomial environments, is well known for its special capacity to develop resistance by selecting chromosomal mutations (i.e. acquired impermeability) or by transferable plasmid enzymes (e.g. extended spectrum carbapenemase or β-lactamase-type).

In Romania, multidrug resistance has become a frequently occurring situation for invasive strains of *P. aeruginosa,* with resistance levels much higher than those encountered in other European countries. In 2012 it was quantified as 51.11% (95% CI 37–65%), and in 2013 it reached 55.8% (95% CI 45.3–65.8%), as opposed to 13% in Europe as a whole. Carbapenem resistance (imipenem and/or meropenem) was at 61.36% (95%CI 46.6–74.3%) in 2012, and at 63.6% (95% CI 53.2–72.9%) in 2013, as compared with around 17% in Europe as a whole. Similarly, piperacillin/tazobactam, ceftazidime, fluoroquinolones, aminoglycosides have higher resistance levels than those encountered in other EU countries [19, 20].

The primary aim of the present study was to investigate the relationship between ICU local resistance bacterial patterns and antibiotic consumption as a basis for future regulations in antibiotic prescribing policies.

## Methods

### Study design

Between 1st January 2012 and 31st December 2013, a prospective study for the monitoring of the antimicrobial resistance (AMR) and of the consumption of antibacterial chemotherapeutic agents was conducted in the largest ICU in western Romania. It is a department with 27 beds, for both surgical and nonsurgical pathologies, in “Pius Branzeu” Emergency Clinical County Hospital in Timisoara, a 1100-bed tertiary care university hospital. During the period of the study, no changes in infection control measures were recorded (i.e. regarding hospital environment decontamination, decontamination/sterilization of instruments and soft materials, promotion of hand hygiene, detection and sterilization of germ carriers among healthcare staff or antimicrobial stewardship interventions).

### Data collection

For the present study, data was collected from the electronic databases of the Microbiology Laboratory and the Pharmacy Department. The approval of the Hospital Ethics Committee was requested and Granted: No. 44346/11.12.2012. The study was based on microbiological and pharmacological surveillance data, with no reference to patients’ personal data or individual medical evolution, and it did not include any supplementary clinical and diagnostic procedure. Therefore, there was no need for patients’ informed consent.

### Sampling

All patients admitted to the ICU over the study period who received antibiotic treatment were included. Patients with an ICU stay of less than 1 h were excluded.

### Variables

The consumption of antimicrobial drugs included antibacterial substances (J01 code of the Anatomical Therapeutic Chemical) and tuberculosis specific drugs (J04). Anti-fungal drugs (J02) and antiviral medication (J05) were excluded. Antibiotic therapy included both empirical treatments and those guided by antimicrobial susceptibility testing (AST).

Consumption was strictly quantified for the period of ICU stay, in Defined Daily Dose (DDD)/1000 patient-days, according to the method established by the WHO Collaborating Centre for Statistical Pharmacologic Methodologies (WHO ATC/DDD Index 2015). DDD is an internationally acknowledged unit of measure representing the average daily dose of antimicrobials administered to an adult weighing 70 kg [21].

### Microbiological methods

Bacterial identification and AST were performed using the Vitek 2 automated system (bio-Mérieux, Marcy-L’Etoile, France). Susceptibility categories were designed according to the CLSI 2012. *P. aeruginosa* ATCC 27853, *Escherichia coli* ATCC 25923, *Staphylococcus aureus* ATCC 25922 reference strains were used as controls in the AST.

A clone strain was defined as a strain of the same bacterial species, with the same antibiotic susceptibility pattern, isolated in the same patient during a 1 month period, regardless of the biological product in which it was isolated. Clone strains were excluded to avoid duplication. Combined-resistant *P. aeruginosa* was considered a strain with resistance to three or more antimicrobial groups among ceftazidime, antipseudomonas penicillins, fluoroquinolones and aminoglycosides. Carbapenem resistance was defined as acquired resistance to at least one agent from this antimicrobial category. The incidence density of resistant strains was defined as the number of resistant strains per 1000 patient-days.

### Statistical analysis

Monthly data regarding the consumption of antibacterial drugs as well as the incidence density for the main resistance phenotypes was aggregated quarterly. The assumption of normal distribution for numerical variables was tested using the Shapiro–Wilk test. Each antibiotic prescription and resistance series was independently explored for trend over time by linear regression. Whenever a statistically significant trend was found (p ≤ 0.05; R^2^ > 0.3), we further analysed possible associations between resistance and antibiotic consumption in paired cross-correlation (with quarterly time lags between − 6 and 6) and linear lagged regression. The approach consisted of step-wise forward modeling, starting with initial single-predictor (i.e. crude) models based on the identified significant trends in resistance phenotypes (as outcomes) and the prescription of antibacterial compounds (as predictors), to which additional variables covering time-lagged consumption and resistance were added. The assessment of the regression models was based on the overall statistical significance, multiple R^2^, its proximity to adjusted R^2^, and the Akaike information criterion to discourage overfitting.

All reported probability values were two-tailed and a 0.05 level of significance was considered to be appropriate. The statistical analysis of the database was performed using the SPSS version 20.0 (IBM, Chicago, IL) and the R version 3.2.3 packages.

## Results

During the period of the study, 17,236 patient-days were cumulated (8466 in 2012 and 8770 in 2013), with a global rate for hospital bed use of 87.44%. The quarterly consumption of antibacterial drugs over the 2 years is presented in Table 1. Tables 2 and 3 show the trends of the main resistance phenotypes during this period. Corroboration of data in Tables 1 and 3 shows that prescription of cephalosporins, fluoroquinolones and aminoglycosides remained constant, while the consumption of penicillins, carbapenems and other antibiotic classes (including aminophenols, imidazole derivatives, glycylglycines, glycopeptides, lincosamides, monobactams, macrolides, anti-Mycobacterium drugs, polymyxins, oxazolidinones, tetracyclines, and sulphonamides) increased. In terms of individual preparations, a significant increase was observed in the consumption of piperacillin associated with tazobactam (gradient = 10.91, R^2^ = 0.698, p = 0.010) and meropenem (gradient = 14.63, R^2^ = 0.753, p = 0.005), respectively, while ceftriaxone consumption showed a sharp decrease (gradient -28.23, R^2^ = 0.681, p = 0.012).

**Table 1:** Quarterly consumption of representative antibiotics and antibacterial compound classes

Time	DDD/1000 patient-days													
	Penicillins	Cephalosporins	Carbapenems	Fluoroquinolones	Aminoglycosides	Other	Piperacillin + tazobactam	Ampicillin + enzyme inhibitors	Amoxicillin + enzyme inhibitors	Ceftriaxone	Cefuroxime	Imipenem + enzyme inhibitor	Meropenem	Ertapenem
Quarter I 2012	27.66	352.71	184.89	72.38	12.44	218.81	27.66	0.00	0.00	341.92	2.37	66.01	64.70	54.18
Quarter II 2012	36.99	265.39	183.44	81.03	29.42	242.46	10.69	18.33	7.96	222.05	35.42	58.73	74.24	50.47
Quarter III 2012	151.70	266.49	245.93	126.71	27.72	319.52	13.40	122.35	8.01	199.17	52.86	93.63	92.09	60.20
Quarter IV 2012	60.22	231.60	159.33	77.81	23.55	246.94	21.00	39.22	0.00	191.74	36.19	67.68	77.88	13.76
Quarter I 2013	118.27	184.84	222.13	73.13	8.29	273.84	53.35	50.48	7.53	99.17	73.45	64.67	113.67	43.79
Quarter II 2013	110.80	148.78	210.68	80.47	21.77	346.61	32.67	60.68	13.63	81.03	55.82	70.31	90.73	49.64
Quarter III 2013	138.16	232.97	270.24	77.04	25.98	339.51	71.43	57.50	8.76	137.83	63.18	55.34	172.22	42.67
Quarter IV 2013	139.90	241.57	286.46	88.46	40.49	468.93	102.33	36.05	0.00	127.11	17.71	97.50	165.75	23.21

**Table 2:** Quarterly incidence density for the main resistance phenotypes

Time	Resistant strains/1000 patient-days						
	MRSA	ESBL *E. coli*	ESBL *K. pneumoniae*	ESBL *P. mirabilis*	Combined-resistant *P. aeruginosa* ^a^	Carbapenem-resistant *P. aeruginosa*	Carbapenem-resistant *A. baumannii*
Quarter I 2012	8.94	0.53	7.89	3.68	1.05	2.10	7.36
Quarter II 2012	4.53	0.91	5.89	3.17	0.00	0.00	4.53
Quarter III 2012	10.57	1.38	10.57	5.97	3.68	4.14	7.81
Quarter IV 2012	4.13	2.29	8.26	5.05	0.92	1.38	6.42
Quarter I 2013	8.76	0.92	2.77	2.77	3.23	3.69	7.84
Quarter II 2013	7.60	0.95	5.23	3.33	2.85	3.80	5.70
Quarter III 2013	5.02	1.83	7.76	1.83	5.48	5.48	5.02
Quarter IV 2013	10.41	1.74	8.68	4.34	6.51	6.94	6.51

^a^Resistance to three or more antimicrobial groups among ceftazidime, antipseudomonas penicillins, fluoroquinolones and aminoglycosides

**Table 3:** Trends in the prescription of antibacterial compounds and for the main resistance phenotypes

	Gradient per quarter	Gradient (95% CI)	*R* ^2^	p value	Trend
Penicilins*	14.61	(0.85; 28.26)	0.529	0.041	↑
Piperacillin + tazobactam*	10.91	(3.74; 18.08)	0.698	0.010	↑
Ampicillin + enzyme inhibitor	3.27	(− 11.14; 17.67)	0.049	0.599	↔
Amoxicillin + enzyme inhibitor	0.34	(− 1.72; 2.39)	0.026	0.702	↔
Cephalosporins	− 15.95	(− 34.73; 2.82)	0.419	0.083	↔
Ceftriaxone*	− 28.23	(− 47.55; − 8.91)	0.681	0.012	↓
Cefuroxime	3.48	(− 5.60; 12.56)	0.128	0.385	↔
Carbapenems*	13.12	(0.48; 25.75)	0.518	0.044	↑
Imipenem + enzyme inhibitor	1.55	(− 4.57; 7.68)	0.060	0.558	↔
Meropenem*	14.63	(6.25; 23.00)	0.753	0.005	↑
Ertapenem	− 3.06	(− 8.76; 2.63)	0.224	0.237	↔
Fluoroquinolones	− 0.60	(− 7.81; 6.60)	0.007	0.844	↔
Aminoglycosides	1.74	(− 1.96; 5.44)	0.180	0.294	↔
Other*	27.90	(10.37; 45.44)	0.717	0.008	↑
MRSA	0.10	(− 0.96; 1.16)	0.009	0.824	↔
ESBL *E. coli*	0.12	(− 0.08; 0.33)	0.261	0.196	↔
ESBL *K. pneumoniae*	− 0.08	(− 1.05; 0.90)	0.006	0.850	↔
ESBL *P. mirabilis*	− 0.15	(− 0.66; 0.37)	0.074	0.515	↔
Combined-resistant P. aeruginosa*	0.78	(0.27; 1.28)	0.707	0.009	↑
Carbapenem-resistant Ps. aeruginosa*	0.74	(0.21; 1.27)	0.666	0.013	↑
Carbapenem-resistant *A. baumannii*	− 0.10	(− 0.59; 3.99)	0.039	0.641	↔

* Results where R^2^ > 0.3 and p ≤ 0.05

It is worth noting that, while the incidence density of methicilin resistant *S. aureus* (MRSA), ESBL producing *E. coli*, *K. pneumoniae*, *Proteus mirabilis* and carbapenem-resistant *A. baumannii* strains remained constant, strains of combined-resistant *P. aeruginosa* (gradient = 0.78, R^2^ = 0.707, p = 0.009) and carbapenem-resistant *P. aeruginosa* (gradient = 0.74, R^2^ = 0.666, p = 0.013) were isolated more frequently.

**Fig. 1:**
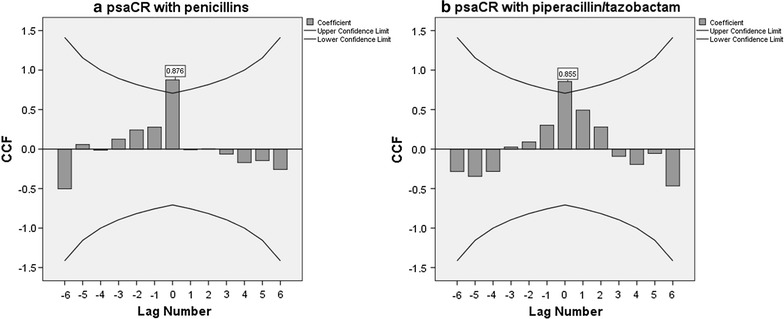
Cross-correlation coefficients between consumption of **a** penicillins/**b** piperacillin–tazobactam and the incidence of combined-resistant *P. aeruginosa* strains

**Fig. 2:**
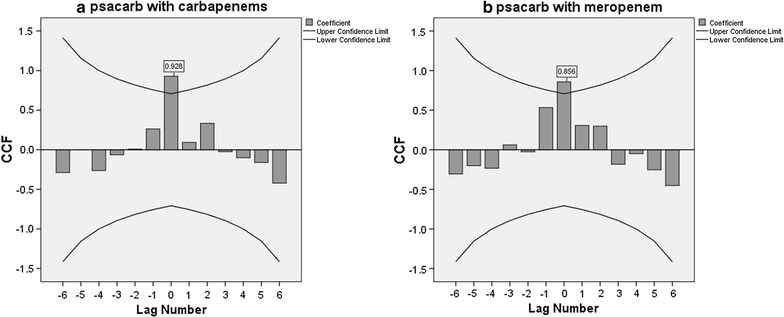
Cross-correlation coefficients between consumption of **a** carbapenems/**b** meropenem and the incidence of carbapenem-resistant *P. aeruginosa* strains

Checks were performed to establish whether a relationship existed between the increase in the number of combined-resistant *P. aeruginosa* and the increased consumption of penicillins and piperacillin/tazobactam, by cross-correlation and construction of four regression models. The same method was applied for carbapenem-resistant *P. aeruginosa* strains, in connection with increased prescription of carbapenems, and meropenem. The cross-correlation coefficients are presented in Figs. 1 and 2.

Table 4 shows the linear regression models for the incidence density of combined-resistant *P. aeruginosa* and the carbapenem-resistant *P. aeruginosa* strains. In each of the four models, the incidence density of the resistant strains is highly dependent upon the current quarter consumption of the specific antibacterials: all the four initial models have high multiple R-squared coefficients, with very significant p values. When adjusted for the consumption of the antibacterial drugs during the previous quarter and/or the resistant *P. aeruginosa* strains in the previous trimester, the first and fourth models showed marked improvements.

**Table 4:** Linear regression models for the incidence density of combined-resistant and carbapenem-resistant *P. aeruginosa* strains

Model			Multiple R^2^ (Adj R^2^)	Model p value	Coefficients estimate ± StdErr	Coefficients p value	AIC change p value
1. PsaCR = f(penicillins)							
	1.1	PsaCR ~ penicillins	0.766 (0.727)	0.004	Penicillins = 0.040 ± 0.009	0.004	
	1.2	PsaCR ~ penicillins + Penlag-1	0.848 (0.773)	0.023	Penicillins = 0.046 ± 0.01 Penlag-1 = 0.012 ± 0.009	0.012	0.0495
						0.255	
	1.3	PsaCR ~ penicillins + PsaCRlag-1	0.920 (0.880)	0.006	Penicillins = 0.046 ± 0.01 PsaCRlag-1 = 0.451 ± 0.171	0.004	0.0039
						0.058	
M	1.4	PsaCR ~ penicillins + PsaCRLlag-1 + Penlag-1	0.953 (0.906)	0.017	Penicillins = 0.047 PsaCRlag-1 = 0.913 ± 0.354 Penlag-1 = − 0.0196 ± 0.013	0.006	0.0066
						0.082	
						0.243	
2. PsaCR = f(piperacillin + tazobactam)							
M	2.1	PsaCR ~ PipTazo	0.730 (0.686)	0.007	PipTazo = 0.061 ± 0.015	0.007	
	2.2	PsaCR ~ PipTazo + PipTazolag-1	0.743 (0.614)	0.066	PipTazo = 0.063 ± 0.024 PipTazolag-1 = − 0.011 ± 0.037	0.060	0.256
						0.783	
	2.3	PsaCR ~ PipTazo + PsaCRlag-1	0.776 (0.664)	0.050	PipTazo = 0.070 ± 0.021 PsaCRlag-1 = − 0.32 ± 0.383	0.031	0.132
						0.451	
	2.4	PsaCR ~ PipTazo + PsaCRlag-1 + PipTazolag-1	0.777 (0.555)	0.166	PipTazo = 0.069 ± 0.027 PsaCRlag-1 = − 0.352 ± 0.517 PipTazolag-1 = 0.006 ± 0.047	0.087	0.862
						0.545	
						0.911	
3. PsaCARB = f(carbapenems)							
M	3.1	PsaCARB ~ carbapenems	0.861 (0.838)	< 0.001	Carbapenems = 0.046 ± 0.008	< 0.001	
	3.2	PsaCARB ~ carbapenems + Carblag-1	0.899 (0.848)	0.010	Carbapenems = 0.046 ± 0.008 Carblag-1 = 0.013 ± 0.01	0.005	0.256
						0.266	
	3.3	PsaCARB ~ carbapenems + PsaCARBlag-1	0.893 (0.839)	0.012	Carbapenems = 0.046 ± 0.009 PsaCARBlag-1 = 0.242 ± 0.208	0.006	0.203
						0.310	
	3.4	PsaCARB ~ carbapenems + PsaCARBlag-1 + Carblag-1	0.899 (0.798)	0.053	Carbapenems = 0.046 ± 0.009 PsaCARBlag-1 = 0.051 ± 0.495 Carblag-1 = 0.010 ± 0.024	0.018	0.975
						0.925	
						0.692	
4. PsaCARB = f(meropenem)							
	4.1	PsaCARB ~ meropenem	0.733 (0.689)	0.0076	Meropenem = 0.046 ± 0.011	0.007	
	4.2	PsaCARB ~ meropenem + Merolag-1	0.794 (0.691)	0.043	Meropenem = 0.039 ± 0.016 Merolag-1 = 0.020 ± 0.018	0.071	0.130
						0.330	
	4.3	PsaCARB ~ meropenem + PsaCARBlag-1	0.738 (0.607)	0.069	Meropenem = 0.052 ± 0.017 PsaCARBlag-1 = − 0.117 ± 0.37	0.037	0.435
						0.765	
M	4.3	PsaCARB ~ meropenem + PsaCARBlag-1 + Merolag-1	0.921 (0.842)	0.037	Meropenem = 0.041 ± 0.011 PsaCARBlag-1 = − 0.709 ± 0.32 Merolag-1 = 0.047 ± 0.018	0.038	0.030
						0.116	
						0.078	

M—best model, selected based on the overall statistical significance, multiple R^2^ (Adjusted R^2^), and Akaike information criterion. For each outcome incidence density model, the first *.1 is the initial model to which additional variables were subsequently added; the Chi square test was applied to check the statistical significance in the Akaike information criterion (AIC) change, compared to the initial model

Carbapenems: carbapenems consumption during the current quarter; Carblag-1: carbapenems consumption during the previous quarter; Merolag-1: meropenem consumption during the previous quarter; Meropenem: meropenem consumption during the current quarter; Penicillins: penicillins consumption during the current quarter; Penlag-1: penicillins consumption during the previous quarter; PipTazo: piperacillin + tazobactam consumption during the current quarter; PipTazolag-1: piperacillin + tazobactam consumption during the previous quarter; PsaCARB: incidence density of carbapenem-resistant *P. aeruginosa* strains during the current quarter; PsaCARBlag-1: incidence density of carbapenem-resistant *P. aeruginosa* strains during the previous quarter; PsaCR: incidence density of combined-resistant *P. aeruginosa* strains during the current quarter; PsaCRlag-1: incidence density of combined-resistant *P. aeruginosa* strains during the previous quarter

The model providing the most accurate picture of the relation between combined-resistant *P. aeruginosa* strains and penicillins consumption during the current quarter is the one which includes both the consumption and the incidence of combined-resistant strains during the previous quarter (multiple R^2^ = 0.953, p = 0.017). When considering only prescriptions of piperacillin/tazobactam during this period, the zero time lag consumption proved decisive (multiple R^2^ = 0.730, p = 0.007), and subsequent adjustments for the previous trimester consumption and/or resistance were not statistically significant.

Carbapenem-resistant *P. aeruginosa* strains correlated most closely to carbapenems consumption during the same quarter (multiple R^2^ = 0.861, p < 0.001). Subsequent adjustments of the initial model improved the multiple R^2^, but not significantly. Both imipenem and ertapenem prescriptions remained constant over the study period, while meropenem consumption increased. The model offering the best explanation of carbapenem resistance of *P. aeruginosa* strains based on meropenem consumption during the current quarter proved to be the adjusted model taking into consideration both the previous consumption and the incidence density of strains during the previous quarter (multiple R^2^ = 0.921, p = 0.037).

## Discussion

During the 2-year period of the study, the consumption of most antibacterial classes remained constant. Cephalosporins, fluoroquinolones and aminoglycosides presented a stable trend, while penicillins, carbapenems and other classes of antimicrobials showed an increasing trend. The consumption of β-lactams with β-lactamase inhibitors increased in the context of the augmented prescription of piperacillin/tazobactam, and a similar pattern was recorded for carbapenems following meropenem consumption, despite the significantly higher price.

Dramatic increase of carbapenems consumption, a consequence of increased incidence of ESBL producing GNB strains, has been associated with the occurrence of carbapenem-resistant *K. pneumoniae* and *A. baumannii* in European ICUs [22–24].

In the hospital in the study, the specific association between the β-lactams with β-lactamase inhibitors prescription and the significant decrease of ceftriaxone consumption can be partly explained by the availability (beginning in 2012) of laboratory diagnoses of *Clostridium difficile* enterocolitis by Enzyme-Linked Fluorescent Assay (with the detection of toxins A and B in fresh stool samples). We noted an increased awareness for *C. difficile* associated diseases (CDAD), not only in the ICU included in the present study, but also at the hospital level (IIIrd generation cephalosporins being the most frequently recorded in treatment protocols of CDAD patients) [25]. The increase for other classes of antibiotics was particularly marked in the context of intensified administration of Colistin, an indirect indication of increased incidence of carbapenem-resistant strains.

In the hospital where the study was undertaken, piperacillin/tazobactam is included in antibiotic therapy protocols for secondary peritonitis (after perforation of cavity organs, appendix, diverticulum), pancreatic abscess, complicated pyelonephritis, but also for infected post-traumatic or post-surgical wounds, especially in gastrointestinal and genital surgery. Meropenem is administered especially for central nervous system (CNS) infections (e.g. acute bacterial meningitis, post-traumatic/post-neurosurgical meningitis, by infection of the ventriculo-peritoneal shunt, etc.) and for ventilator-associated pneumonia/septicemies with lung starting point. The increased consumption of the two chemotherapeutic agents may be explained by a 52.08% increase in the number of neurosurgery patients and a 31.05% increase in general surgery cases in 2013 as compared with 2012.

During the studied period, the incidence density of the main resistance phenotypes remained constant. On the other hand, MDR *P. aeruginosa* strains (both combined-resistant and carbapenem-resistant) showed statistically significant increasing trends.

In this study, the cross-correlation analysis identified very strong positive correlation between consumption of penicillins and piperacillin/tazobactam, and combined-resistant *P. aeruginosa* strains, respectively, each for the current quarter. An even stronger positive correlation was identified between the consumption of carbapenems and the incidence of carbapenem-resistant *P. aeruginosa*. A similar positive correlation has been identified by Jaggi et al. between consumption and carbapenem resistance of *Acinetobacter* and *Pseudomonas* strains [26]. The same authors found a positive correlation between the consumption and piperacillin/tazobactam resistance for *Acinetobacter* strains, but a negative correlation in the case of *Pseudomonas* [26].

A series of other studies found strong correlations between carbapenems consumption and resistance in non-fermentative strains [27–30], with a decrease in the incidence of these strains consecutive to decreased consumption [31].

Regarding the fitted model of the combined resistance of *P. aeruginosa* strains and penicillins prescription, a striking finding was the high value of the regression coefficient for previous quarter resistance (though just marginally significant from the statistical point of view), which shows the importance of endemic bacterial clones and suggest this is an issue which deserves further study. The regression models prove the dependence of the current quarter resistance of *P. aeruginosa* strains on the consumption of antibacterial drugs in that quarter, and in the previous one, as well as on the incidence of resistant strains circulating in that particular hospital department over the previous quarter.

One study from China found that the quarterly prevalence of carbapenem-resistant *P. aeruginosa* strains was strongly correlated, with a time lag, with the quarterly use of anti-pseudomonal carbapenems (p = 0.001). On the other hand, no correlation was identified with other classes of antibacterial drugs (anti-pseudomonal beta-lactam/beta-lactamase inhibitors, aminoglycosides and fluoroquinolones) [15].

In the short term, the results of this investigation have led to changes in local policies for antibiotic prescription, in two areas. On the one hand, we intensified the measures aimed at reducing the selection pressure: an antibiotic prescription form was implemented, an infectious disease specialist was employed to supervise antibiotic treatments, physicians were trained in the importance of de-escalation and reduced period of antibiotic prophylaxis. In order to avoid empirical treatments, a GeneXpert^®^ Instrument Systems was acquired. On the other hand, in order to reduce the incidence density of resistant strains, screening for carriers of MDR strains upon hospital admission became mandatory, ICU isolation facilities were expanded, and a campaign was organized to improve healthcare staff compliance with the hand hygiene policy.

There are limitations in this study: other antimicrobial resistance risk factors (e.g. causes of hospitalization, readmission rates) were not assessed, and no analysis was undertaken of the entry of new sources of MDR strains, which could have functioned as possible supplementary confounders.

## Conclusions

During the observed period, the incidence of combined-resistant and carbapenem-resistant *P. aeruginosa* strains increased significantly, paralleling the increase in consumption of β-lactams with β-lactamase inhibitors (piperacillin/tazobactam) and carbapenems (meropenem). The cross-correlation coefficients and the fitted regression models convey additional evidence that resistance during a given quarter depends on the consumption of antibacterial chemotherapeutic drugs in that quarter, but also consumption during the previous quarter, combined with the incidence of circulating resistant strains.

## Additional files

**Additional file 1.** The consumption of antibacterial drugs.

**Additional file 2.** The resistance phenotypes.

## Abbreviations

AMR: antimicrobial resistance
AST: antimicrobial susceptibility testing
CDAD: *Clostridium difficile* associated diseases
CNS: central nervous system
DDD: defined daily dose
ESBL: extended spectrum β-lactamase
GNB: Gram-negative bacilli
ICU: intensive care unit
MDR: multidrug-resistant
MRSA: methicilin resistant *Staphylococcus aureus*
